# Diagnosis and Treatment Difficulty in Early-Onset Peristomal Pyoderma Gangrenosum Associated With Ulcerative Colitis: A Case Report

**DOI:** 10.7759/cureus.22405

**Published:** 2022-02-20

**Authors:** Ken Imaizumi, Hiroyuki Kasajima, Kazutoshi Terashima, Naoe Furukawa, Kazuaki Nakanishi

**Affiliations:** 1 Gastrointestinal Surgery, Hakodate Municipal Hospital, Hakodate, JPN; 2 Gastroenterological Surgery, Hakodate Municipal Hospital, Hakodate, JPN; 3 Wound, Ostomy and Continence Nursing, Hakodate Municipal Hospital, Hakodate, JPN

**Keywords:** early-onset, ostomy care, ulcerative colitis, inflammatory bowel disease, pyoderma gangrenosum, peristomal pyoderma gangrenosum

## Abstract

Peristomal pyoderma gangrenosum (PPG) is a rare dermatological condition associated with gastroenterological disease. Most gastrointestinal surgeons find it difficult to suspect and treat PPG, especially at early onset. The patient was an 18-year-old female. The patient underwent three-stage restorative proctocolectomy for refractory ulcerative colitis. On postoperative day (POD) 9, the trocar wound near the ileostomy site dehisced. Because the wound culture was positive, the wound was treated with an antibacterial agent as an infection. However, the wound worsened. The patient was referred to a dermatologist for diagnosis. PPG was diagnosed on POD 37. Wound management was initiated using topical steroids. The wound caused difficulties in pain and dressing management. Although infliximab was administered as a systemic therapy, it was discontinued because of allergic symptoms. Sealing therapy with hydrofiber dressing and adequate stoma pouching with stoma paste provided good exudate absorption and a clean environment by protecting the wound from stoma excretion. Oral prednisone was initiated on POD 82. Improvement in the wound condition was observed with a prednisone dose of 30 mg/day. Complete remission was achieved seven months after onset. Twelve months after the surgery, stoma closure was performed. The local cutaneous condition remained in remission without exacerbation. Suspicion of PPG can be difficult when it develops early after stoma creation. We never forget that PPG should be suspected when a progressive ulcerative lesion is found around the stoma, even early after operation. If PPG is suspected, a multidisciplinary team plays an essential role in its diagnosis and management.

## Introduction

Pyoderma gangrenosum (PG) is a rare extraintestinal manifestation of inflammatory bowel disease (IBD). Peristomal PG (PPG) is a subtype of PG that occurs at surgical placement around a stoma. There have been few reports of PPG. Most gastrointestinal surgeons rarely encounter PPG. Hence, they find it difficult to suspect PPG, especially at early onset. Furthermore, therapeutic management requires not only classic PG treatment, but also a stoma-care technique. Here, we report a case of PPG after restorative proctocolectomy for ulcerative colitis (UC).

## Case presentation

**Figure 1:**
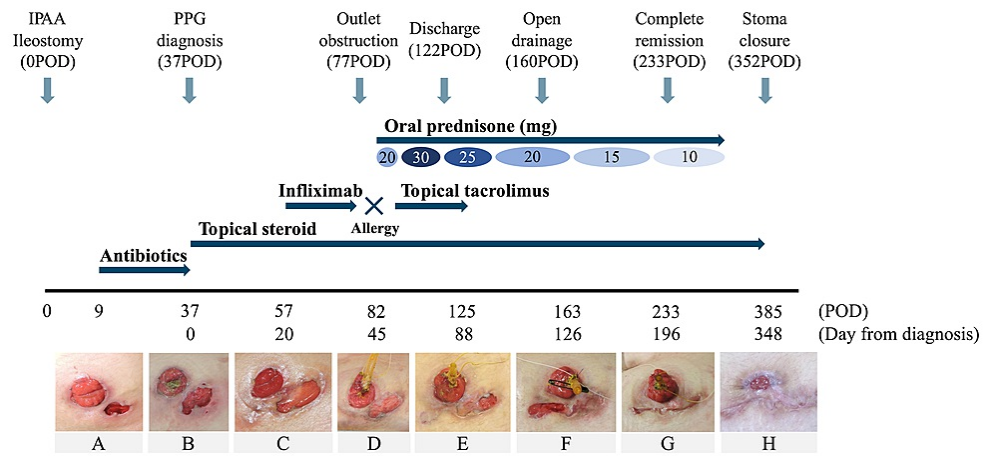
Patient's clinical course with photographic progression and therapeutic management A, Appearance of PPG. B, Diagnosis of PPG. C, Initiation of systemic therapy. D, Stoma outlet obstruction. E, Discharge. F, Open wound pocket drainage. G, Complete remission. H, After stoma closure. IPAA, ileal pouch anal anastomosis; POD, postoperative day; PPG, peristomal pyoderma gangrenosum.

The patient was an 18-year-old female. Her preoperative body mass index (BMI) was 22.8 kg/m^2^. The patient was diagnosed with refractory UC. The patient had not developed any extraintestinal manifestations. The patient underwent laparoscopic total colectomy and an end ileostomy as the first stage of the three-stage surgery. After four months, the patient underwent laparoscopic proctectomy and ileal pouch anal anastomosis with diverting loop ileostomy as the second stage of the three-stage surgery. On postoperative day (POD) 9, the trocar wound near the stoma dehisced (Figure 1A). 

**Figure 2:**
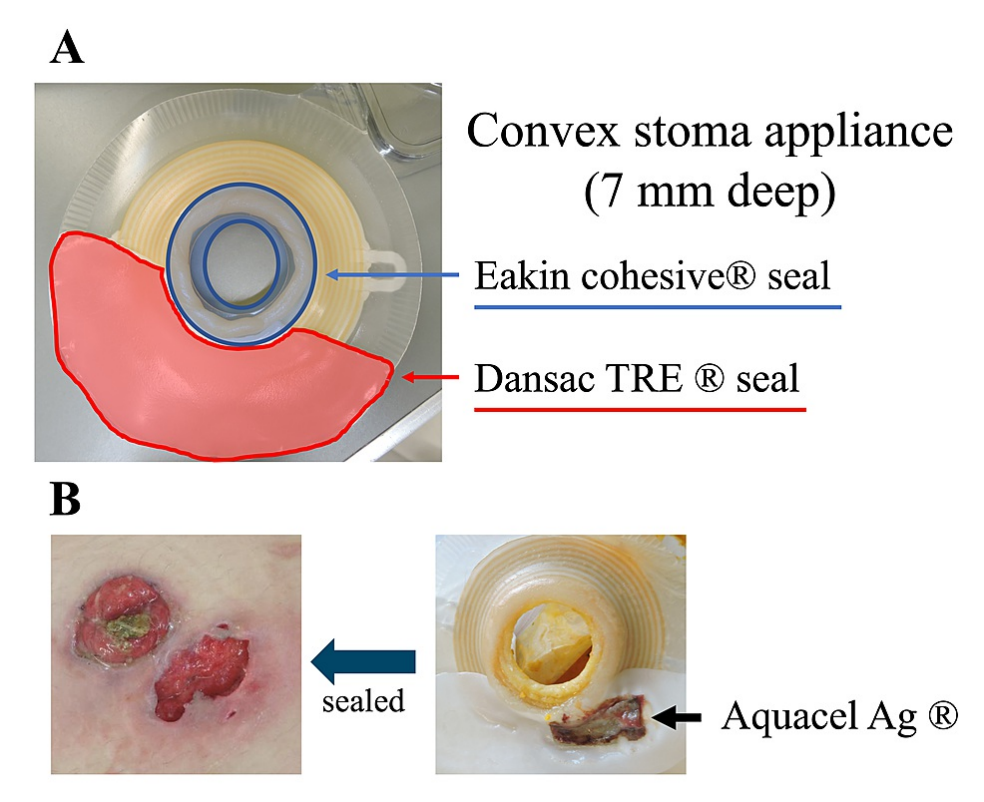
Stoma-care technique and sealing therapy A, Devising stoma pastes for a convex stoma appliance. The blue and red areas indicate pasting Eakin cohesive® seal and Dansac TRE® seal, respectively. B, Sealing therapy with hydrofiber dressing.

Since Gram-positive cocci, Gram-negative rods, and polymorphonuclear cells were cultured from the wound, the wound was cleaned and treated with an antibacterial agent as a surgical site infection. However, the wound showed ulceration with erythema and pain. The patient was referred to a dermatologist for diagnosis. On POD 37, the wound was diagnosed as a PPG (Figure 1B). Topical steroids were started for PPG treatment. First, ointment agents (betamethasone valerate and betamethasone butyrate) were selected. A lotion agent (mometasone furoate) was injected when a pocket was formed under the skin. The wound caused difficulties in pain and dressing management. A hydrofiber dressing with silver (Aquacel Ag®) was used for wound care. On POD 57 (Figure 1C), infliximab was administered as systemic therapy, in consultation with the gastroenterologist, because systemic steroids were resistant to UC treatment. However, infliximab was discontinued during the second course because of allergic symptoms. On POD 77, stoma outlet obstruction occurred. A decompression tube was inserted into the stoma (Figure 1D). Because the stoma output increased in addition to the location of the wound where the stoma pouch was covered, the stoma pouch leaked frequently and the maintenance of local cleanliness was complex. Certified wound, ostomy and continence (WOC) nurses (KT and NF) for skin and excretion care devised stoma appliance choice and fitting. The wound was managed by devising stoma pastes (e.g. Eakin cohesive® seal and Dansac TRE® seal) for a convex stoma appliance (7 mm deep). Sealing therapy with hydrofiber dressing and adequate stoma pouching with stoma paste provided good exudate absorption and a clean environment by protecting the wound from stoma excretion (Figure 2). 

Oral prednisone was initiated on POD 82. Topical 0.1% tacrolimus was supported for the treatment. Improvement in the wound condition was observed with a prednisone dose of 30 mg/day (Figure 1E). After the wound pocket was opened, the amount of oral steroids gradually reduced while confirming that the local condition had not worsened (Figure 1F). Complete remission (CR) was achieved seven months after onset, and the oral steroid was terminated one month after CR (Figure 1G). Twelve months after the second-stage surgery, stoma closure was performed. The local cutaneous condition remained in remission without exacerbation (Figure 1H).

## Discussion

PPG is a rare dermatological condition based on gastroenterological diseases. There have been limited case series and few retrospective studies related to PPG [1-6]. When PPG occurs early in the postoperative course after stoma creation, diagnosis and treatment may be difficult for gastrointestinal surgeons. This patient’s course revealed the difficulty of PPG diagnosis and treatment decision making at early onset. 

Among IBD patients who undergo stoma surgery, 2-4% develop PPG [2,7,8]. According to a systematic review, IBD was seen in 80% of cases of PPG, which were composed of 50% and 30% in Crohn’s disease and UC, respectively [9]. In addition to IBD, malignant diseases and trauma were involved [9]. Previous report has demonstrated that female sex, higher BMI, and use of pouch belts are risk factors for developing PPG [6]. In terms of stoma type, ileostomy and end-stoma were more relevant to PPG than colostomy and loop-stoma, respectively [9]. In the present case, a female patient and ileostomy were matched. The median duration between stoma formation and PPG diagnosis was reported to be 101.5 days (range 14-2641 days) [5]. Some cases of PPG have an early onset, whereas others have a late onset. In particular, when PPG occurs at an early onset after stoma creation as in the present case, the suspicion of PPG will be difficult and late because surgeons initially suspect postoperative complications (e.g. surgical site infection). The diagnosis of PPG is clinically decided and requires exclusion of other causes. In 2018, expert consensus defined the diagnostic criteria for classic PG (Table 1) [10].

**Table 1:** Diagnostic criteria for classic pyoderma gangarenosum In addition to the major criterion, patients must have at least four minor criteria to meet the diagnostic criteria. Source of the table in Reference 10. ^a^Including histologically indicated stains and tissue cultures. ^b^Ulcer should extend past the area of trauma.

Type	Diagnostic criteria
Major criterion	Biopsy of ulcer edge demonstrating a neutrophilic infiltrate
Minor criteria	
Histology	Exclusion of infection^a^
History	Pathergy (ulcer occurring at sites of trauma)^b^
	Personal history of inflammatory bowel disease or inflammatory arthritis
	History of papule, pustule, or vesicle that rapidly ulcerated
Clinical examination (or photographic evidence)	Peripheral erythema, undermining border, and tenderness at site of ulceration
	Multiple ulcerations (at least one occurring on an anterior lower leg)
	Cribriform or “wrinkled paper” scar(s) at sites of healed ulcers
Treatment	Decreasing ulcer size within one month of initiating immunosuppressive medication(s)

However, diagnostic criteria for PPG have not yet been established. Although skin biopsy is recommended to rule out malignancy and vasculitis, biopsy is not essential for diagnosis because the histopathological characteristics of PPG are nonspecific. According to previous reports, biopsy has been performed in only about half of the cases [9]. We believe that urgent consultation with a dermatologist is important if PPG is clinically suspected. Misdiagnosis and subsequent inappropriate treatment of PPG can lead to adverse outcomes.

Various therapeutic options are available for PPG treatment. Treatment typically starts with wound care and topical agents such as corticosteroids or calcineurin inhibitors. However, few patients achieved CR in PPG with topical therapy alone. Wang et al. reported CR rates of 13% and 14% for topical calcineurin inhibitors and steroids, respectively [6]. Therefore, although topical agents are often used as the first-line treatment for PPG because of common side effects, systemic therapies or surgical interventions may be required to achieve CR. Systemic steroids and anti-TNF (tumor necrosis factor) agents have been described as effective therapeutic options for PPG. Especially, anti-TNF agents including infliximab and adalimumab induced CR in 63% of patients in a previous cohort study [6]. In this report, systemic therapy should be selected depending on comorbid conditions [11]. Our patient initially received infliximab for systemic steroid resistance in UC. Systemic corticosteroids were mainly used as critical agents in a systematic review [9]. This therapy achieved CR in 52% of patients [9]. Compared with classic PG, surgical interventions, including stoma closure and resection of active IBD, have proven effective in managing PPG [3,6,9]. The present case did not recur after stoma closure. However, it is unclear whether stoma closure should be performed when the PPG is poorly controlled. In contrast, PPG recurrence is common after stoma relocation [3]. Surgical relocation of the stoma for PPG treatment is generally not recommended [9]. In any case, because the consensus and guidelines of PPG treatment have not been established, trial and error of different therapies is often necessary for the treatment. Wound care and ostomy appliances provided by stoma care nurses are critical for promoting wound healing and preventing skin irritation. The healing time to achieve complete resolution has been reported to be approximately 5-10 months [5,6]. Most PPG cases resolve with treatment, although recurrence was seen in 20% of patients within a year [6].

## Conclusions

This report suggests that suspicion of PPG can be difficult when it develops early after stoma creation. We never forget that PPG should be suspected when a progressive ulcerative lesion is found around the stoma, even early after operation. If PPG is suspected, a multidisciplinary team (the dermatologist, WOC nurse, etc.) plays an essential role in its diagnosis and management.
